# Combined Effect of Different Flower Stem Features on the Visiting Frequency of the Generalist Ant *Lasius niger:* An Experimental Study

**DOI:** 10.3390/insects12111026

**Published:** 2021-11-14

**Authors:** Elena V. Gorb, Stanislav N. Gorb

**Affiliations:** Department of Functional Morphology and Biomechanics, Zoological Institute, Kiel University, Am Botanischen Garten 9, 24098 Kiel, Germany; sgorb@zoologie.uni-kiel.de

**Keywords:** calcium carbonate coverage, cuffs, cuticular folds, epicuticular wax projections, greasy pole syndrome, slaked (hydrated) lime, nectar robbing, *Smyrnium rotundifolium*

## Abstract

**Simple Summary:**

Flowering plants usually attract insect pollinators by offering them nectar, pollen or other energetically valuable sources. To deter ants, which are unreliable pollinators and can act as nectar thieves, plants have developed different systems either inside the flowers or associated with the stems. The latter one, called *greasy pole syndrome*, is based on the combined effect of several stem features hampering the access of ants to the apically located flowers. In this study, we examined the effects of different flower stem features in the round-leaved Alexanders *Smyrnium rotundifolium* on the visiting frequency of the generalist ant species, the black garden ant *Lasius niger*. We conducted the experiments with ants running on dry wooden sticks mimicking four different types of stems. To attract ants, we placed a sweet sugar syrup droplet on a stick tip. Ants visited different types of stem-mimicking sticks with significantly different frequencies. The highest number of insects were registered on untreated stick samples, whereas the lowest visiting frequency was observed on sticks bearing cuff-like structures (serving as macroscopic physical barriers) covered with a nano/microparticle film, which caused the slipperiness of the surface. Thus, by combining macroscopic obstacles and slippery surfaces, plants can protect their flowers from undesirable crawling visitors such as ants.

**Abstract:**

In order to understand the effects of the morphology and surface texture of flower stems in *Smyrnium rotundifolium* on the visiting frequency of generalist ants, we conducted experiments with *Lasius niger* ants running on dry wooden sticks mimicking different types of stems: (1) intact (grooved) sticks; (2) sticks painted with slaked (hydrated) lime (calcium carbonate coverage) imitating plant epicuticular wax coverage; (3) intact sticks with smooth polyester plate-shaped cuffs imitating upper leaves; and (4) intact sticks bearing cuffs painted with slaked lime. Ants were attracted by the sweet sugar syrup droplets placed on a stick tip, and the number of ants visiting the drops was counted. Our data showed significant differences in the visiting frequencies between the different types of stem-mimicking samples. The number of recorded ants progressively decreased in the following order of samples: intact sticks—painted sticks—sticks with intact cuffs—sticks with painted cuffs. These results clearly demonstrated that micro/nanoscopic surface coverages and macroscopic physical barriers, especially if combined, have a negative impact on the attractiveness of stems to ants. This study provides further evidence for the hypothesis that having a diversity of plant stems in the field, generalist ants prefer substrates where their locomotion is less hindered by obstacles and/or surface slipperiness.

## 1. Introduction

To attract insect pollinators, mostly from the orders Hymenoptera, Diptera, Lepidoptera, and Coleoptera, the angiosperms (flowering plants) usually produce and offer them nectar, pollen or other energetically valuable resources that are in the main associated with flowers [1]. On the other hand, plants have also developed different systems, primarily inside the flowers, such as toxic nectar or chemical/morphological modifications of the floral tissues, in order to deter ants [2,3,4,5,6,7,8,9] that generally belong to the floral antagonists, which are unreliable pollinators and can act as nectar thieves [10,11]. In addition to consuming nectar, ants sometimes attack and deter legitimate pollinators, in that way decreasing pollination success [7,12,13,14,15,16]. The *greasy pole syndrome* is another defense mechanism preventing ants from visiting flowers and robbing nectar [17,18,19,20]. It was described in plants from the genera *Salix* (Salicaceae*), Hypenia* and *Eriope* (both Lamiaceae) and is based on the combined effect of several non-floral stem features hampering the access of ants to the apically located reproductive organs of plants. These features include slender elongate erect stems, rigid spreading trichomes (i.e., hair-like protuberances extending from the epidermis of aerial plant tissues) on the lower internodes, three-dimensional epicuticular wax coverage (composed of projections of hydrophobic cuticular lipids deposited onto the aerial primary surfaces of higher plants) and often swellings in the upper internodes [19]. Admittedly, the trichomes deter ground foraging ants, whereas the waxes prevent ants from climbing the upper stem portions. The stem shape is presumably responsible for its extreme motility in windy conditions, and the fistulose swellings provide additional physical barriers hampering ant locomotion.

Until now, the main attention was given to the contribution of the wax coverage on stems to ant deterrence. First, Kerner von Marilaun [17] detected the presence of wax on *S. daphnoides* stems and suggested its hampering effect on ants trying to reach the nectar-bearing flowers. Later field observations on *Eriope* plants showed that ants of all sizes failed to climb up these stems covered with microscopic wax projections [18]. Simple experiments with *H. vitifolia* and *Lasius niger* (Hymenoptera: Formicidae) demonstrated that ants were not able to gain a foothold on the waxy stems, perhaps because they dislodged wax projections and therefore fell to the ground [19]. Our previous experiments, where we recorded the traversed distances of *L. niger* ants on the waxy flower stems of *Anethum graveolens* (Apiaceae)*, Dahlia pinnata* and *Tagetes patula* (both Asteraceae) clearly showed that ants were able to walk significantly lower distances on the wax-covered stems compared to the reference wax-free ones [21]. Ants moved much more slowly and carefully on wax-covered stems; however, in all the tests performed, not a single ant fell down from either surface. The later study on the frequency of plant visits by *L. niger* ants was performed with five plant species bearing different surface structures on their stems: *Alchemilla mollis* (Rosaceae), with wax projections and long thread-shaped trichomes; *Lilium lancifolium* (Liliaceae), without wax, but with ribbon-shaped trichomes and cuticular folds (i.e., surface microstructures usually caused by the folding of the cuticle over the outer cell wall of epidermal cells); *Salvia nemorosa* (Lamiaceae), without wax, but with trichomes of various lengths and cuticular folds; *Tulipa gesneriana* (Liliaceae), with wax projections and lacking trichomes; *Paeonia lactiflora* (Paeoniaceae), having neither of the above surface features [22]. It was found that, on the one hand, ants avoided climbing the wax-covered stems, especially if trichomes were lacking. On the other hand, some trichome-bearing stems having specific trichome micromorphologies were also ignored by ants. The last result is in line with that of the only previous study on the contribution of the effect of trichomes to the greasy pole syndrome [19], showing that *L. niger* ants, which had been introduced at the ground level, became entangled in the trichomes near the base of the *H. vitifolia* stem.

**Figure 1:**
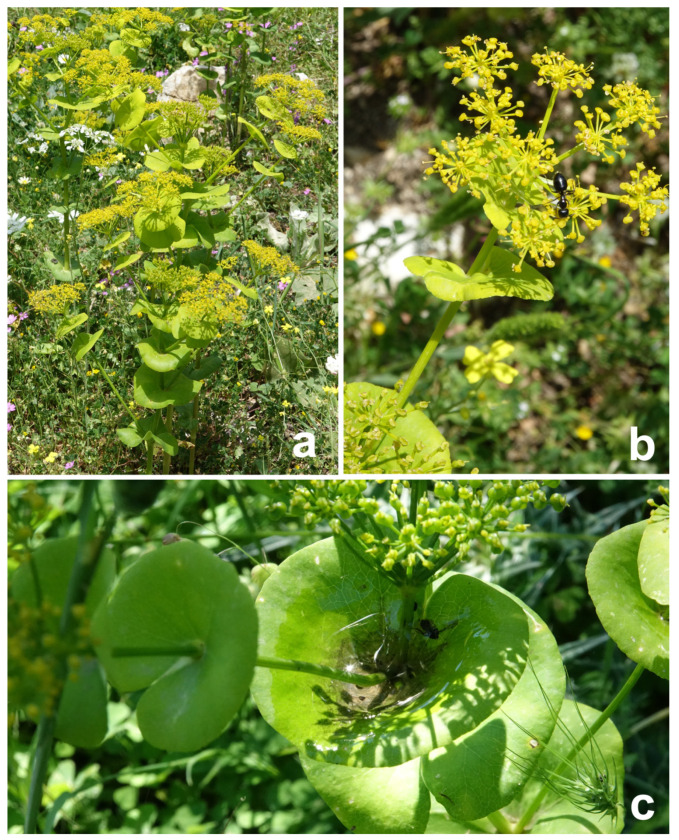
The plant *Smyrnium rotundifolium*. (**a**) General view of the plant in the natural habitat. (**b**) Branched umbel with an ant foraging on flowers. (**c**) Upper leaves forming a kind of cuff around flower stems. Note a water pool visible in the cuff located in the center of the picture.

Whereas the impact of the waxes and trichomes covering the stems of plants showing the greasy pole syndrome on ant deterrence has been previously examined, the effect of macroscopic characteristics (e.g., the swellings in the upper internodes mentioned above) has not been studied so far. In the present study, besides the microscopic surface features, we are also focusing on the macroscopic stem structures, which can potentially serve as a physical barrier for ants—the sessile upper leaves clasping the stem like cuffs in the round-leaved Alexanders *Smyrnium rotundifolium* (Apiaceae) (Figure 1c). In this plant, the small flowers that are assembled in branched umbels (Figure 1b) are exposed and openly offer, like a cafeteria, nectar and pollen for pollinators. However, ants are also readily attracted by the nectar (Figure 1b). To ensure visitation from only winged insects, purposely so to be more easily cross-pollinated by them and to avoid the visits of nectar-robbing ants, this plant has developed a system of different characteristics, both macroscopic (cuffs formed by upper leaves) and microscopic (cuticular folds and three-dimensional epicuticular wax coverage), on their flower stems, hindering the attachment and locomotion of ants.

In order to understand the effects of the flower stem morphology and surface texture in *S. rotundifolium* on the visiting frequency of generalist ants, we conducted the experiment with *L. niger* ants and dry wooden sticks mimicking different types of plant stems, among them that of *S. rotundifolium*. Ants were attracted by the sweet sugar syrup droplets placed on a stick tip, and the number of ants visiting the droplets on the different stick samples was counted. Our zero hypothesis was that the frequency of ant visits should be similar for all the stick sample types tested. We have found that both the microscopic and macroscopic stem characteristics negatively impact ant visits, and if combined, have a much more pronounced effect. The obtained results support the previously proposed hypothesis [22] that having a diversity of plant stems in the field, generalist ants prefer surfaces where their attachment and locomotion are less hindered by the stem features.

## 2. Materials and Methods

### 2.1. Plants and Insects

The round-leaved Alexanders *S. rotundifolium* Mill., also treated at the rank of the subspecies under *S. perfoliatum* (*S. perfoliatum* subsp. *rotundifolium* (Mill.) Hartvig) [23] or even as a variety (*S. perfoliatum* var. rotundifolium (Mill.) Fiori) [24], is an erect, branching perennial [25] of 30–60 cm height, with a ridged, but wingless, not hollow stem and broadly oblong, divided (biternate), green lower (basal and in the lower stem portion) leaves (Figure 1a). Bright, rounded upper leaves, being actually the bracts, have more or less entire margins, are sessile and clasp the stem (Figure 1c). Tiny yellow flowers assembled in branched umbels, which have up to 12 rays (Figure 1b), are produced in late spring and early summer (description after [26,27]). The plants are common in olive groves, thickets and in dry locations [26] and occur in the central and eastern Mediterranean regions at an elevation of up to 800 m [28]. No specialized ant species associated with *S rotundifolium* has been reported so far.

The black garden ant *L. niger* L. is an omnivorous species that originated from South America and Africa [29] but is recently spread over Europe, where it became widely distributed at sites of human disturbance (e.g., roadsides, gardens, etc.). Workers of this ant species regularly forage on diverse plants [30], can rob nectar from flowering plants and collect honeydew from aphids living on plants. We selected *L. niger* as a model generalist ant species for experiments because (1) it is associated with a variety of plant species (personal observations) and (2) it was available in a great number at the study site. Attachment system of *L. niger* is composed of paired claws and a pad-like, smooth arolium on each foot (for details see [21]).

### 2.2. Microscopy

Plant material for cryo-scanning electron microscopy (SEM) examination was collected from the plants growing in the olive grove near the Gardiki Castle (surroundings of Agios Matthaios, Corfu, Greece, 39.476500° N, 19.884983° E). Upper leaves and upper portions of the stem were cut off from living plants and kept for 24 h inside small plastic vials containing wet paper in order to prevent desiccation of the plant material. Then, small samples (1 cm × 1 cm) from the middle region of the leaves and from the stem were cut out, attached mechanically to a small vice on a metal holder and frozen in a cryo-stage preparation chamber at −140 °C (Gatan ALTO 2500 Cryo Preparation System, Gatan Inc., Abingdon, UK). Frozen samples of the upper (adaxial) and lower (abaxial) leaf sides and of the stem surface were sputter coated with gold–palladium (thickness 6 nm) and examined in a frozen condition in a cryo-SEM Hitachi S-4800 (Hitachi High-Technologies Corporation, Tokyo, Japan) at 3 kV accelerating voltage and −120 °C temperature. Types of wax projections were identified according to Barthlott et al. [31].

To visualize the surface textures of dry wooden sticks, transparent polyester film and calcium carbonate coverage used in the experiment with ants (see Section 2.3), small pieces of the intact stick and polyester foil as well as painted stick and cuff (ca. 1 cm^2^) were sputter coated with gold–palladium (6 nm thickness) and studied in the SEM at 3 kV accelerating voltage and room temperature (ca. 20 °C).

Morphometrical variables of surface features were measured from digital images using SigmaScan Pro 5 software (SPSS Inc., Chicago, IL, USA). These data are presented in the text as mean ± SD for *n* = 10.

### 2.3. Experiment

**Figure 2:**
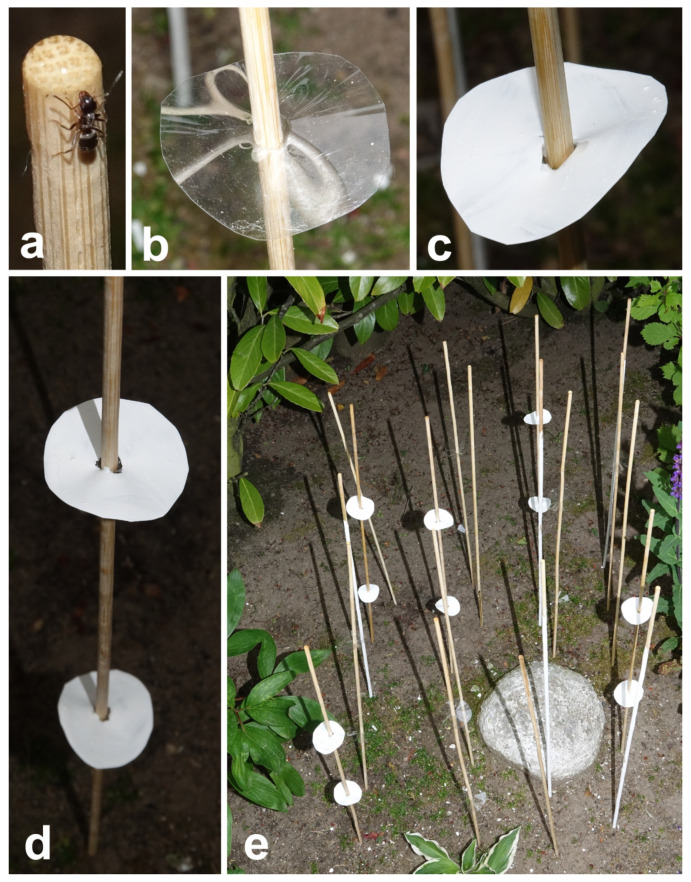
Set-up for the experiment with ants. (**a**) An ant feeding on a sweet droplet deposited on the stick tip. (**b**) Transparent cuff made out of transparent polyester film. (**c**) The cuff bearing calcium carbonate coverage that mimics an epicuticular plant wax. (**d**) Two cuffs places on a stick sample. (**e**) Arrangement of test sticks at the study site.

For the experiment with ants, instead of native plant stems of *S. rotundifolium,* we used dry wooden sticks (50 cm long, 0.3 cm in diameter, with grooved surface; Gardol Splittstab; BAHAG AG, Mannheim, Germany) dug to a 5 cm depth into the soil. We prepared four types of sticks mimicking different types (i.e., having diverse morphologies and surface textures) of plant stems: (1) intact, untreated sticks; (2) sticks painted with slaked, or hydrated, lime, thereby imitating plant epicuticular wax coverage; (3) untreated sticks with smooth plate-shaped cuffs imitating upper leaves (Figure 2b); and (4) untreated sticks bearing cuffs painted with slaked lime (Figure 2c). The cuffs were prepared in the following way: discs (ca. 5 cm in diameter) with a small hole in the center were cut out from the transparent polyester film (color laser transparency film 5752341, 125 μm thick; Office Depot, La Venio, the Netherlands) and firmly attached to sticks at the heights of 15 and 30 cm from the ground level (two cuffs per stick) (Figure 2d). To get wax-mimicking coverage on the surface, we painted sticks/cuffs with slaked lime (calcium hydroxide Ca(OH)_2,_ Art-Nr. KK03.1; Carl Roth GmbH, Karlsruhe, Germany) dissolved in double-distilled water in the ratio 1:1 (lime to water). After drying under air conditions (in the presence of carbon dioxide CO_2_), the limewater resulted in the formation of a micro/nanostructured layer of calcium carbonate (CaCO_3_) on the surface.

The experiment was carried out in a private garden (Kronshagen, Schleswig-Holstein, Germany; 54°20′11.354″ N, 10°5′15.547″ E). Five sticks of each type (altogether 20 sticks) were placed at nearly similar distance (ca. 10 cm) from each other; the order of different stick types was randomized (Figure 2e). This approach allowed us a direct comparison of samples located at a very narrow space (ca. 1 m^2^), and therefore exposed to approximately the same number of foraging ants. The position of each stick was changed every day during the experiment. A small droplet (ca. 15 µL) of the customly prepared sugar syrup (double-distilled water to sugar weight ratio was 1:1) was deposited on the stick tip. After 12 h, each single stick sample was individually checked, and the number of ants feeding on the syrup droplet (Figure 2a) was registered. After counting ants, the droplets were renewed. The experiment was performed during 14 dry days (336 h) in June (from 10 to 23 of June) at a temperature of 20–25 °C and 40–65% relative humidity. We counted ants twice a day, in the morning (at 8:00) and in the evening (at 18:00–22:00); on some weekend days (15.06, 16.06, and 22.06), and additionally during the daytime (at 11:00–16:00). In all, 31 recordings on each single stick sample were made and the visits of 470 ants were registered.

To determine whether there is a significant difference between the expected frequencies and the observed frequencies of ant visits in different stick types, the number of ant visits per stick type was tested using the Chi-square test (software Good Calculator, free online calculators). Pairwise multiple comparisons between the stick types were performed using the Chi-square test with Bonferroni correction (α = 0.05/6 = 0.0083). For each test, the expected frequencies in each stick type were calculated as expected = total number of registered ants/number of stick types.

## 3. Results

### 3.1. Micromorphology of Plant Surfaces

**Figure 3:**
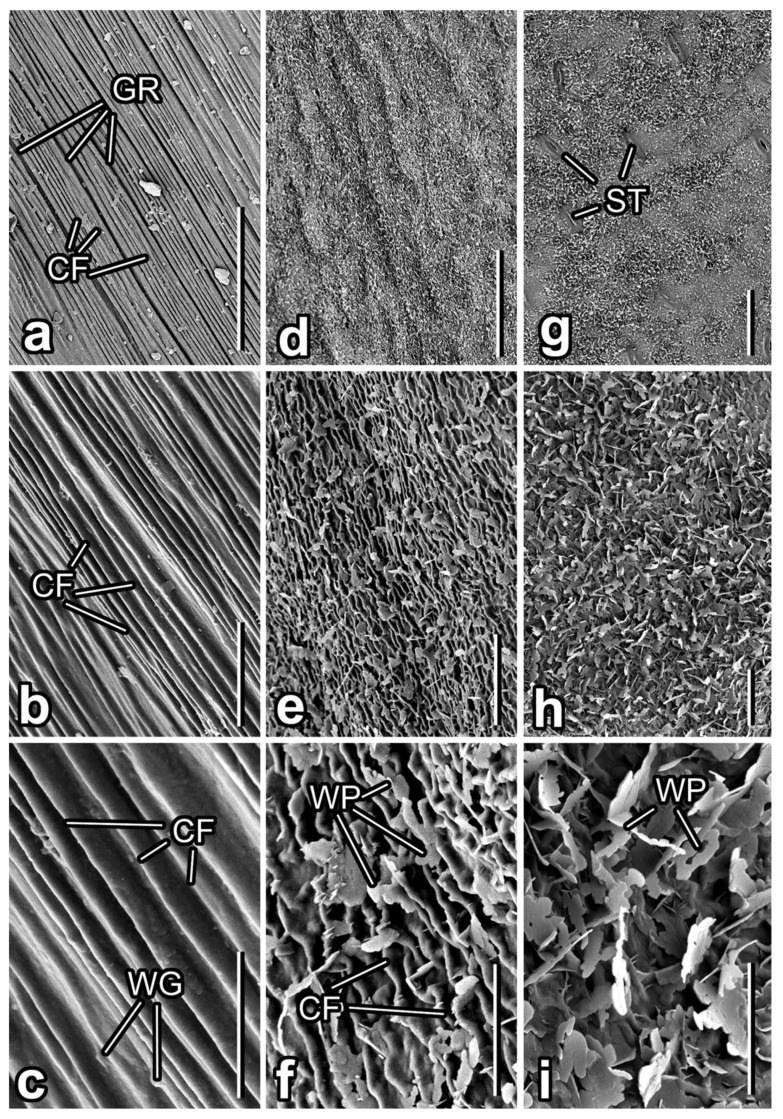
Surfaces of different organs in the *Smyrnium rotundifolium* plant (cryo-SEM). (**a**–**c**) Flower stem. (**d**–**f**) Upper (adaxial) leaf side. (**g**–**i**) Lower (abaxial) leaf side. Abbreviations: CF—cuticular folds; GR—grooves; ST—stomata; WG—wax granules; WP—wax platelets. Scale bars: 50 µm (**a**,**d**,**g**), 10 µm (**b**,**e**,**h**), 5 µm (**c**,**f**,**i**).

The flower stem surface of *S. rotundifolium* is noticeably structured with rather uniform, straight cuticular folds (width: 1.68 ± 0.34 µm; height: 0.68 ± 0.16 µm) running parallel along the longitudinal stem axis (Figure 3). Usually, each 5–10 (7.2 ± 1.3) folds build clusters separated through somewhat wider and deeper grooves (Figure 3a). On top, cuticular folds bear numerous, nearly rounded nanostructures (diameter: 235 ± 73 nm), apparently epicuticular wax granules (Figure 3c).

The adaxial leaf side is slightly uneven because of the convex shape of the epidermal cells (Figure 3d), and has a prominent hierarchical microstructure composed of a dense network of winding cuticular folds and scattered (occurrence: ca. 0.5 µm^−2^) epicuticular wax projections (Figure 3e). The folds, being relatively short, narrow and shallow (length: 3.80 ± 1.13 µm; width: 0.53 ± 0.07 µm; height: 0.39 ± 0.06 µm), are responsible for the irregular corrugate surface appearance (Figure 3f). Small (length: 1.17 ± 0.26 µm; width: 0.57 ± 0.19 µm; thickness: 0.05 ± 0.01 µm) flat wax projections (irregular platelets) having highly variable shapes and non-entire margins protrude from the cuticle at various, often acute, angles and do not show any characteristic orientation (Figure 3f).

The abaxial leaf side bears stomata having smooth guard cells (Figure 3g) and a multilayered, extremely dense (occurrence in the external layer: ca. 2 µm^−2^) coverage of flat, often interconnected epicuticular wax projections (membranous platelets) in the regions between the stomata (Figure 3h). These platelets with irregular margins or even filiform extensions vary greatly in shape and size (length: 2.00 ± 0.36 µm; width: 0.79 ± 0.16 µm; thickness: 0.04 ± 0.01 µm) (Figure 3i). They have neither specific orientation nor distinct arrangement on the surface.

### 3.2. Microtexture of Samples Used in the Experiment

**Figure 4:**
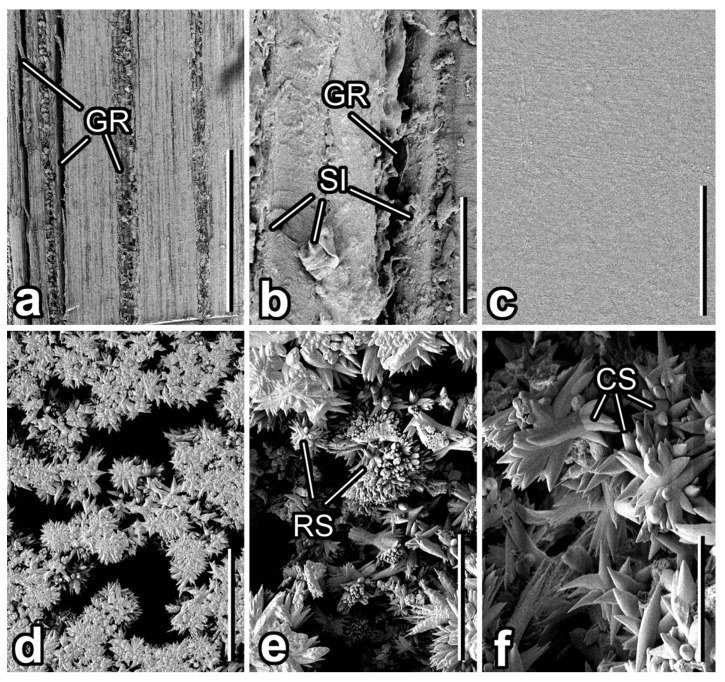
Surfaces of different experimental samples (SEM). (**a,b**) Intact stick. (**c**) Transparent polyester film. (**d**–**f**) Calcium carbonate (CaCO_3_) coverage. Abbreviations: CS—cone-shaped structures; GR—grooves; RS—double rosettes; SI—surface irregularities. Scale bars: 1 mm (**a**), 20 µm (**d**), 10 µm (**b**,**e**), 5 µm (**c**,**f**).

The surface of the wooden sticks has straight grooves running parallel along the longitudinal stick axis (Figure 4a). Both the width and the height/depth of both the grooves and especially the elevations (groove width: 5.64 ± 4.14 µm; elevation width: 23.74 ± 14.69 µm) vary greatly at different stick portions. Moreover, non-uniform, often flake-like microscopic (1–4 µm) irregularities having various dimensions (2.31 ± 1.27 µm in length/diameter) protrude from the surface (Figure 4b). On the contrary, the polyester film surface is rather smooth at the microscopic scale (Figure 4c).

Calcium carbonate coverage obtained from an aqueous solution of calcium hydroxide after air drying represents a rather loose layer (Figure 4d) composed of double rosettes of numerous (more than ten in each rosette) accrete, solid, cone-shaped microscopic (length: 1.82 ± 0.46 µm) structures (Figure 4e,f). The cones have either a sharp or rounded tip (however, the same within one individual rosette) and the varying ratio of cross section diameters between the basal, middle, and upper parts (basal: 0.53 ± 0.18 µm; middle: 0.53 ± 0.18 µm; upper: 0.23 ± 0.05 µm). This variability gives different tapered shapes of structures ranging from the relatively slender to almost barrel-like. The voids between the rosettes and between the cones within a rosette are responsible for the high porosity of the material that the coverage is made of.

### 3.3. Ant Visits to Different Samples

Data on the number of ant individuals visiting each sample during the experimental time (31 recordings per sample) are presented in Supplementary Materials (Table S1). All the sticks were visited at least twice (out of 31 recordings) by ants. The maximum number (12) of ant individuals seen at the same time was detected on the intact stick, followed by 10 ants recorded on both the intact and the painted stick samples. On the stick type with transparent cuffs, a maximum four ants at once were detected, whereas on sticks with painted cuffs, not more than two individuals at the same time were registered.

**Figure 5:**
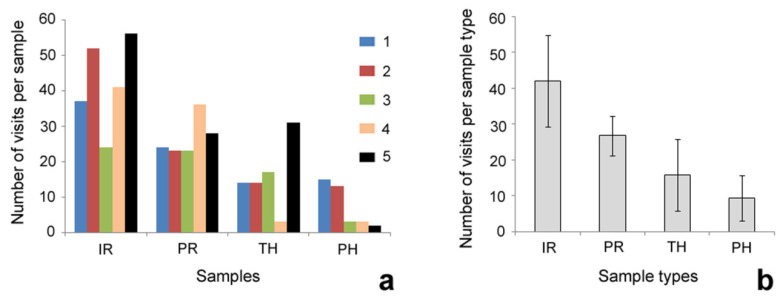
Number of ant visits per sample (**a**) and per sample type (mean and SD) (**b**). Abbreviations: IR—intact sticks; PH—sticks with painted (i.e., bearing calcium carbonate coverage) cuffs; PR—painted sticks bearing calcium carbonate coverage; TH—sticks with unpainted, transparent cuffs; 1, 2, 3, 4, 5—different individual sticks in each sample type.

In Figure 5, the number of registered ant individuals (counted during all 31 recordings) for each stick and each sample type is shown. The number of recorded ants progressively decreased in the following order of samples: intact sticks—painted sticks—sticks with intact cuffs—sticks with painted cuffs (Figure 5b). Ants demonstrated clear, significantly different preferences (χ^2^ = 129.5084, d.f. = 3, *p* < 0.0001). Most preferable samples were intact sticks that differed significantly from all other (modified) types of sticks (intact sticks vs. painted sticks: χ^2^ = 102.9845, *p* < 0.0001; intact sticks vs. intact cuffs: χ^2^ = 59.1793, *p* < 0.0001; intact sticks vs. painted cuffs: χ^2^ = 16.7906, *p* < 0.001, Chi-square test with Bonferroni correction (α = 0.05/6 = 0.0083), d.f. = 1). Moreover, the modified stick types differed significantly from each other (painted sticks vs. intact cuffs: χ^2^ = 8.1270, *p* < 0.005; painted sticks vs. painted cuffs: χ^2^ = 41.5934, *p* < 0.0001; intact cuffs vs. painted cuffs; χ^2^ = 14.1402, *p* < 0.001, Chi-square test with Bonferroni correction (α = 0.05/6 = 0.0083), d.f. = 1). 

## 4. Discussion

Despite the world abundance of ants, very few plant species (ten so far) are described as ant-pollinated, and ants are regarded as undesirable flower visitors that are only capable of nectar thieving [10]. Moreover, ants can scare pollinators away and even assail them [7,12,13,14,15,16]. To avoid such negative effects caused by ants on pollination, many plants have evolved different chemical/morphological floral adaptations [2,3,4,5,6,7,8,9] or a complex of morphological stem features preventing the access of ants to the flowers (i.e., greasy pole syndrome) [17,18,19,20,21,22], especially if flowers are openly placed and bear nonhidden (i.e., freely accessible) nectaries, like in the round-leaved Alexanders *S. rotundifolium* studied here.

*Smyrnium rotundifolium* possesses several stem- and leaf-related macroscopic and micro/nanoscopic characteristics, such as clusters of microscopic cuticular folds decorated with nanoscopic epicuticular wax projections on the flower stems and the cuffs, formed by the upper leaves bearing either wax projections on the adaxial side or a combination of cuticular folds and wax projections on the abaxial side. In our experiments, in order to evaluate the effect of different features on the frequency of ant visits, we used different types of wooden sticks mimicking stems with different characteristics: intact sticks with a grooved surface imitating stems with grooves between the clusters of cuticular folds; painted sticks imitating stems with wax projections; sticks with transparent polyester cuffs mimicking upper leaves; and sticks with painted cuffs imitating upper leaves bearing wax projections.

Our experimental results clearly demonstrated that both the macroscopic structures and the micro/nanoscopic surface coverages significantly reduced the visiting frequency of ants. In the first case, the upper leaves, completely wrapping the stems and making the cuffs, represent a physical barrier where ants have to overcome three transitions: (1) from the vertical stem to the adaxial side of the cuff (ceiling situation) [32,33]; (2) from the adaxial to abaxial side of the cuff; and (3) from the abaxial side of the cuff to the vertical stem. The first two transitions are particularly complicated, since insects can easily lose the grip there and fall down. Moreover, the cuffs usually collect rain and dew water and form a kind of pool (see such a pool in Figure 1c), creating an additional physical barrier for ants to overcome. In our study, we did not test the effect of water pools experimentally, but observed their presence in nature. However, it has been also previously shown for one ant species, *Camponotus schmitzi* (Hymenoptera: Formicidae), living in symbiosis with the Bornean pitcher plant, *Nepenthes bicalcarata* (Nepenthaceae), that it can swim and dive in the pitcher’s fluid to forage for food, but compared to running, the swimming involved lower stepping frequencies and larger phase delays within the legs of each tripod [34]. Even though this kind of behavior is known for this particular ant species, it seems that these ants are strongly specialized in this symbiosis for their diving behavior in order to get some reward in the form of protein-rich food from the plant. That is why we do not expect that *L. niger* and a majority of other ant species associated with the habitats where *S. rotundifolium* occurs, can overcome the water filled out pools in order to get access to the flower nectar.

Additionally, our experiments (see Figure 5b) showed even lower numbers of ants on the unpainted cuffs (solely the effect of the cuffs) than on the painted sticks (effect of wax). This fact demonstrates the rather strong effectiveness of the cuff-like morphology against nectar-robbing ants.

As for the effect of the cuticular folds, we did not mimic these microscopic surface structures in our experimental samples. In the literature, it has been hypothesized that the folds support the attachment and locomotion of pollinators on flowers [35,36], but recent experimental studies performed with non-pollinating beetles on various leaf and petal surfaces showed a decrease of insect attachment forces on the cuticular folds compared to flat substrates due to the reduction of the real contact area [37,38,39,40]. However, in our previous study on the frequency of plant visits by *L. niger* ants that was performed with five plant species having different surface structures on their stems, *Lilium lancifolium* flower stems covered with both cuticular folds and long soft trichomes showed a rather high number of ant visits [22]. We suggested that in this plant species, the soft twisted trichomes probably “compensate” for an unsuitable quality of the stem surface caused by the cuticular folds.

The wax projections were simulated by calcium carbonate crystals in our experimental samples (painted sticks and painted cuffs). Hydrated lime is one of the first minerals used in “ancient particle film technology” [41], which alone or in mixture represents the prevailing insect-repelling material used in agriculture in the early 1900s [42] and is still presently applied against insect pest and plant pathogens. Whereas in livestock farming it is mostly used for disinfectant purposes, producing a dry and alkaline environment in which bacteria do not readily multiply, in horticultural farming it serves as an insect repellent causing no harm to either pests or plants [43]. In particular, lime-based products have been reported to function as oviposition deterrents for the *Rhagoletis indifferens* fly (Diptera: Tephritidae) [44]. Hydrated lime treatments may also repel insects, and these effects have been confirmed for different insect species [45,46,47]. Our recent experimental study revealed that calcium carbonate coverage greatly diminished the attachment of the bug *Nezara viridula* (Heteroptera: Pentatomidae) due to its distinct microrough surface topology and, to a lesser extent, due to the contaminating effect on the insect adhesive organs [41]. Moreover, the high absorption ability, in particular the water absorption ability, of the calcium carbonate film described in [41] may contribute to insect attachment reduction, as has been previously found for both *Coccinella septempunctata* and *Harmonia axyridis* beetles (both Coleoptera: Coccinellidae) on nanoporous substrates [48,49].

Both stick sample types bearing the calcium carbonate coverage (painted sticks and painted cuffs) showed significantly lower visiting frequencies of ants compared to the corresponding untreated samples (intact sticks and transparent cuffs, respectively). These data are in line with the results of numerous previous experimental studies performed with many insect and plant species (reviewed in [50]), showing that prominent epicuticular wax coverage in plants usually reduces insect attachment using different mechanisms: (1) the reduction of the real contact area between the substrate and the tips of insect attachment organs (the roughness hypothesis); (2) the contamination of insect adhesive organs by the wax projections (the contamination hypothesis); (3) the adsorption of fluid secretion from the insect adhesive pads due to the high capillarity of the wax coverage (the fluid absorption hypothesis); (4) hydroplaning caused by the appearance of a thick layer of fluid caused by the dissolving of the wax material in insect adhesive fluid (the wax dissolving hypothesis); and (5) the formation of a separation layer between the insect attachment organs and the substrate [51,52]. Moreover, our previous studies with *L. niger* ants and the wax-bearing stems of *Anethum graveolens, Dahlia pinnata, Tagetes patula* and *Tulipa gesneriana* showed that ants avoided these stems but were still able to walk on such antiadhesive vertical substrates when they had no other choice [21,22]. It was concluded that the reason why nonspecialized ants usually do not climb wax-covered stems is that the additional locomotory efforts are needed to master climbing on “greasy” stems. In the present study, ants still climbed up the painted sticks and visited, although much more rarely, the painted cuffs. The fact that we still observed ants on these sample types means that ants can hold and walk on a waxy surface. However, very few ant visits detected on the sticks with painted cuffs indicated that if macroscopic obstacles like cuffs are combined with micro/nanoscopic coverages (i.e., waxes in the case of plants and calcium carbonate film in our experimental samples), such substrates become extremely challenging for ant locomotion. Thus, by having flower stems with both macroscopic barriers and prominent micro/nanoscopic coverages, plants protect their flowers bearing openly placed nectaries from undesirable crawling visitors such as ants.

Thus, in contrast to the previous experimental studies on the greasy pole syndrome that were performed either with plants or plant samples [19,21,22], we here for the first time have used artificial samples mimicking different types of plant flower samples. For this, we applied dry wooden sticks instead of the original plant stems and imitated the microscopic surface characteristics and macroscopic obstacles by using calcium carbonate particle coverage and transparent polyester film, respectively. The role of the macroscopic stem structures in plant defense against the generalist ant species was first tested here. We confirmed once more the deterring effect of microscopic surface features on insects [50,51,52] and showed experimentally for the first time a negative influence of the macroscopic barriers on the visiting frequency of ants. We discovered that a combination of microscopic (three-dimensional epicuticular wax coverage) and macroscopic (cuffs formed by upper leaves) stem-related characteristics has a great impact on ant visits: in this way, plants can protect their generative organs from these unwanted creeping visitors. The stem-related characteristics are plant adaptations to avoid the visits of nectar-robbing ants, probably by hindering their attachment and locomotion, in order to ensure the visitation of pollinators.

## 5. Conclusions

Our data show significant differences in ants’ visiting frequencies among the different types of stem-mimicking samples. The results clearly demonstrate that micro/nanoscopic surface structures and macroscopic physical barriers, especially if combined, have a negative impact on the attractiveness of the flower stems to ants. This study provides further evidence for the hypothesis that having a diversity of plant stems in the field, generalist ants prefer substrates where their locomotion is less hindered by obstacles or surface slipperiness [22]. 

## Data Availability

The complete experimental data set supporting reported results is provided in the supplementary Table S1.

## Supplementary Materials

The following is available online at https://www.mdpi.com/article/10.3390/insects12111026/s1, Table S1: Number of ant individuals counted on stick samples mimicking different features of the *Smyrnium rotundifolium* flower stem at the time of 31 visits of experimenters during 14 days.

## References

[1] Waser N.M., Waser N.M., Ollerton J. (2006). Specialization and generalization in plant-pollinator interactions: A historical perspective. Plant-Pollinator Interactions: From Specialization to Generalization.

[2] Van der Pijl L. (1955). Some remarks on myrmecophytes. Phytomorphology.

[3] Janzen D.H. (1977). Why don’t ants visit flowers?. Biotropica.

[4] Guerrant E.O., Fiedler P.L. (1981). Flower defenses against nectar-pilferage by ants. Biotropica.

[5] Junker R.R., Blüthgen N. (2008). Floral scents repel potentially nectar-thieving ants. Evol. Ecol. Res..

[6] Junker R.R., Gershenzon J., Unsicker S.B. (2011). Floral odor bouquet loses its ant repellent properties after inhibition of terpene biosynthesis. J. Chem. Ecol..

[7] Galen C., Cuba J. (2001). Down the tube: Pollinators, predators, and the evolution of flower shape in the alpine skypilot, *Polemonium viscosum*. Evolution.

[8] Tagawa K. (2018). Repellence of nectar-thieving ants by a physical barrier: Adaptive role of petal hairs on *Menyanthes trifoliata* (Menyanthaceae). J. Asia Pac. Entomol..

[9] Villamil N., Boege K., Stone G.N. (2019). Testing the distraction hypothesis: Do extrafloral nectaries reduce ant-pollinator conflict?. J. Ecol..

[10] Peakall R., Handel S.N., Beattie A., Huxley C.R., Cutler D.E. (1991). The evidence for, and importance of, ant pollination. Ant-Plant Interactions.

[11] Willmer P.G., Nuttman C.V., Raine N.E., Stone G.N., Pattrick J.G., Henson K., Stillman P., McIlroy L., Potts S.G., Knudsen J.T. (2009). Floral volatiles controlling ant behaviour. Func. Ecol..

[12] Tsuji K., Hasyim A., Harlion, Nakamura K. (2004). Asian weaver ants, *Oecophylla smaragdina*, and their repelling of pollinators. Ecol. Res..

[13] Ness J.H. (2006). A mutualism’s indirect costs: The most aggressive plant bodyguards also deter pollinators. Oikos.

[14] Lach L. (2008). Argentine ants displace floral arthropods in a biodiversity hotspot. Divers. Distrib..

[15] Hansen D.M., Müller C.B. (2009). Invasive ants disrupt gecko pollination and seed dispersal of the endangered plant *Roussea simplex* in Mauritius. Biotropica.

[16] Cembrowski A.R., Tan M.G., Thomson J.D., Frederickson M.E. (2014). Ants and ant scent reduce bumblebee pollination of artificial flowers. Am. Nat..

[17] Kerner von Marilaun A. (1878). Flowers and their Unbidden Guests.

[18] Harley R.M. (1988). Evolution and distribution of Eriope (Labiatae) and its relatives in Brazil. Proceedings of a Workshop on Neotropical Distributions.

[19] Harley R., Huxley C.R., Cutler D.E. (1991). The greasy pole syndrome. Ant-Plant Interactions.

[20] Juniper B.E., Hamilton R.J. (1995). Waxes on plant surfaces and their interactions with insects. Waxes: Chemistry, Molecular Biology and Functions.

[21] Gorb E., Gorb S. (2011). How a lack of choice can force ants to climb up waxy plant stems. Arthropod-Plant Interact..

[22] Gorb S.N., Gorb E.V. (2019). Frequency of plant visits by the generalist ant *Lasius niger* depends on the surface microstructure of plant stems. Arthropod-Plant Interact..

[23] Strid A. (1986). Mountain Flora of Greece.

[24] Fiori A. (1925). Nuova Flora Analitica d’Italia.

[25] Kiehn M. (2015). Pflanzen mit invasivem Potenzial in Botanischen Gärten X: *Smyrnium perfoliatum* L. (Apiaceae). Carinthia II (Klagenfurt, Austria).

[26] Papiomioglou V. (2006). Wild Flowers of Greece.

[27] Unterarten von Smyrnium perfoliatum. http://www.mittelmeerflora.de/Zweikeim/Apiaceae/smyr_perfoliatum.htm.

[28] Jäger E.J., Reckardt K. (1998). Beiträge zur Wuchsform und Biologie der Gefäßpflanzen des herzynischen Raumes. 2: *Smyrnium perfoliatum* L. (Apiaceae). Hercynia N. F..

[29] Klotz J.H., Hansen L., Pospischil R., Rust M. (2008). Urban Ants of North America and Europe: Identification, Biology, and Management.

[30] Hölldobler B., Wilson E.O. (1990). The Ants.

[31] Barthlott W., Neinhuis C., Cutler D., Ditsch F., Meusel I., Theisen I., Wilhelmi H. (1998). Classification and terminology of plant epicuticular waxes. Bot. J. Linn. Soc..

[32] Ritzmann R.E., Quinn R.D., Fischer M.S. (2004). Convergent evolution and locomotion through complex terrain by insects, vertebrates and robots. Arthropod Struct. Dev..

[33] Bußhardt P., Gorb S.N., Wolf H. (2011). Activity of the claw retractor muscle in stick insects in wall and ceiling situations. J. Exp. Biol..

[34] Bohn H.F., Thornham D.G., Federle W. (2012). Ants swimming in pitcher plants: Kinematics of aquatic and terrestrial locomotion in *Camponotus schmitzi*. J. Comp. Physiol..

[35] Stork N.E. (1980). Experimental analysis of adhesion of *Chrysolina polita* (Chrysomelidae, Coleoptera) on a variety of surfaces. J. Exp. Biol..

[36] Barthlott W., Ehler N. (1977). Raster-Elektronenmikroskopie der Epidermisoberflächen von Spermatophyten. Trop. Subtrop. Pflanzenwelt.

[37] Prüm B., Seidel R., Bohn H.F., Speck T. (2011). Plant surfaces with cuticular folds are slippery for beetles. J. R. Soc. Interface.

[38] Prüm B., Bohn H.F., Seidel R., Rubach S., Speck T. (2013). Plant surfaces with cuticular folds and their replicas: Influence of microstructuring and surface chemistry on the attachment of a leaf beetle. Acta Biomater..

[39] Voigt D., Schweikart A., Fery A., Gorb S. (2012). Leaf beetle attachment on wrinkled surfaces: Isotropic friction on anisotropic surfaces. J. Exp. Biol..

[40] Bräuer P., Neinhuis C., Voigt D. (2017). Attachment of honeybees and greenbottle flies to petal surfaces. Arthropod-Plant Interact..

[41] Salerno G., Rebora M., Piersanti S., Saitta V., Kovalev A., Gorb E., Gorb S. (2021). Reduction in insect attachment caused by different nanomaterials used as particle films (kaolin, zeolite, calcium carbonate). Sustainability.

[42] Glenn D.M., Puterka G.J. (2005). Particle films: A new technology for agriculture. Hort. Rev..

[43] Agricultural Lime. https://en.wikipedia.org/wiki/Agricultural_lime.

[44] Yee W.L. (2012). Behavioural responses by *Rhagoletis indifferens* (Dipt., Tephritidae) to sweet cherry treated with kaolin- and limestone-based products. J. Appl. Entomol..

[45] Barata J.M.S., Santos J.L.F., Da Rosa J.A., Gomes R.D. (1992). Evaluation of triatomine behavior under the effect of contact with calcium hydroxide CaOH_2_: Mortality rates of *Triotoma infestans* and *Rhodnius neglectus* (Hemiptera, Reduviidae). An. Soc. Entomol. Bras..

[46] Boucher J., Adams R., Johnson F., Pachauskas R. (1993). Eggplant: Hydrated lime as an insect repellent. Insectic. Acaricidae Tests.

[47] Strack T., Cahenzli F., Daniel C., Wolfrum S., Heuwinkel H., Wiesinger K., Reents H.J., Hülsbergen K.-J. (2017). Kaolin, lime and rock dusts to control *Drosophila suzukii*. Ökologschen Landbau Weiterdenken: Verantwortung Übernehmen, Vertrauen Stärken.

[48] Gorb E., Hosoda N., Miksch C., Gorb S. (2010). Slippery pores: Anti-adhesive effect of nanoporous substrates on the beetle attachment system. J. R. Soc. Interface.

[49] Gorb E.V., Lemke W., Gorb S.N. (2019). Porous substrate affects a subsequent attachment ability of the beetle *Harmonia axyridis* (Coleoptera, Coccinellidae). J. R. Soc. Interface.

[50] Gorb E.V., Gorb S.N. (2017). Anti-adhesive effects of plant wax coverage on insect attachment. J. Exp. Bot..

[51] Gorb E.V., Gorb S.N. (2002). Attachment ability of the beetle *Chrysolina fastuosa* on various plant surfaces. Entomol. Exp. Appl..

[52] Gorb E.V., Purtov J., Gorb S.N. (2014). Adhesion force measurements on the two wax layers of the waxy zone in *Nepenthes alata* pitchers. Sci. Rep..

