# Supplementary material for: Combined Effect of Different Flower Stem Features on the Visiting Frequency of the Generalist Ant Lasius niger: An Experimental Study
Source: Insects. 2021 Nov 14;12(11):1026. doi: 10.3390/insects12111026 (PMC8623630; doi:10.3390/insects12111026)
Supplement: Supplementary file 1 [file insects-12-01026-s001.zip › insects-1440540-supplementary.pdf]

Table S1. Number of ant individuals counted on stick samples mimicking different features of the *Smyrnium rotundifolium* flower stem at the time of 31 visits of experimenters during 14 days

IR - intact stick (control)

PR- painted stick (with $CaCo_3$ coverage)

TH - stick with unpainted, transparent cuffs

PH - stick with painted cuffs (bearing CaCo3 coverage)

	IR1	PR1	TH1	PH1	IR2	PR2	TH2	PH2	IR3	PR3	TH3	PH3	IR4	PR4	TH4	PH4	IR5	PR5	TH5	PH5
Begin: So 10.06, 20:00																				
Mon 10.06, 8:00	0	1	0	0	0	0	0	0	0	0	0	0	0	0	0	0	1	1	1	0
Mon 10.06, 21:00	0	0	0	0	0	0	0	0	0	0	0	0	0	0	0	0	0	3	1	0
Tue 11.06, 8:00	0	0	0	0	0	0	0	0	0	0	0	0	1	3	0	0	0	2	0	0
Tue 11.06, 21:00	1	0	0	0	1	0	3	0	0	0	0	0	0	1	0	0	1	1	1	0
Wed 12.06, 8:00	1	0	0	0	0	1	1	0	0	0	0	0	0	0	0	0	0	0	1	0
Wed 12.06, 21:00	0	0	0	0	1	0	1	0	0	0	0	0	0	1	0	0	0	0	0	0
Thr 13.06, 8:00	0	0	1	0	1	0	0	0	0	0	0	0	0	1	0	0	0	0	0	0
Thr 13.06, 22:00	0	0	0	0	1	0	0	0	0	10	0	0	0	0	0	0	0	2	0	0
Fri 14.06, 8:00	0	0	1	0	0	1	1	0	0	0	0	0	0	0	0	0	0	0	1	0
Fri 14.06, 19:00	0	0	0	0	0	0	0	0	0	0	1	0	0	0	0	0	0	0	1	0
Sat 15.06, 8:00	0	0	0	0	0	0	0	0	0	0	1	0	0	1	0	0	0	0	1	0
Sat 15.06, 11:00	1	0	0	0	5	3	0	0	1	0	0	0	0	0	0	0	12	5	1	0
Sat 15.06, 22:00	1	1	3	0	3	1	2	0	0	1	2	1	10	3	0	1	2	2	2	1
Sun 16.06, 8:00	0	0	0	0	1	0	0	1	0	0	0	0	0	0	0	0	0	1	0	0
Sun 16.06, 16:00	1	0	1	0	1	1	0	1	0	0	0	0	0	0	0	0	1	0	1	0
Sun 16.06, 19:30	2	0	2	0	0	0	0	0	1	1	0	2	0	2	0	0	7	1	2	0
Mon 17.06, 8:00	1	1	1	0	1	0	1	0	0	0	0	3	0	1	0	0	1	3	1	0
Mon 17.06, 18:00	1	2	1	0	2	1	1	1	3	2	3	1	1	1	1	0	2	2	3	1
Tue 18.06, 8:00	0	1	0	1	2	1	0	0	0	0	0	0	1	1	0	0	0	0	0	0
Tue 18.06, 20:00	1	1	0	1	3	0	0	1	1	0	0	0	2	0	0	0	0	0	0	0
Wed 19.06, 8:00	2	0	0	0	5	1	0	0	2	0	0	0	0	0	0	0	0	0	0	0
Wed 19.06, 18:30	5	1	0	1	0	0	0	1	2	0	0	0	2	1	0	0	5	0	0	0

Thr 20.06, 8:00	4	0	0	0	1	1	0	0	3	0	2	0	3	4	0	0	1	0	0	0
Thr 20.06, 20:00	1	0	0	1	1	1	0	0	0	0	1	0	3	0	0	1	2	0	0	0
Fri 21.06, 8:00	3	1	0	1	0	2	0	0	1	0	1	0	6	3	0	0	3	1	1	0
Fri 21.06, 21:00	1	3	0	3	6	2	0	2	0	0	1	1	2	3	1	0	5	2	1	0
Sat 22.06 8:00	2	3	0	1	4	1	0	0	2	3	2	0	2	3	0	0	3	1	4	0
Sat 22.06 16:00	2	3	0	2	7	1	0	2	2	3	2	2	5	4	0	1	3	0	1	0
Sat 22.06 21:00	1	0	1	1	2	0	0	2	2	0	0	1	0	1	0	0	0	0	2	0
Sun 23.06 8:00	4	4	2	1	3	3	1	1	1	1	1	1	2	1	0	0	2	1	4	0
Sun 23.06 20:00	2	2	1	2	1	2	3	1	3	2	0	2	1	1	1	0	5	0	1	0
SUM for 31 recordings	37	24	14	15	52	23	14	13	24	23	17	14	41	36	3	3	56	28	31	2
